# Neurofibromatosis type 1, fibromuscular dysplasia, and ischemic stroke: an association lost in time? A case report

**DOI:** 10.1590/1677-5449.202201182

**Published:** 2023-05-26

**Authors:** Igor Oliveira da Fonseca, Gustavo José Luvizutto, Isaac Pantaleão Souza, André Petean Trindade, Carlos Clayton Macedo de Freitas, Rodrigo Bazan, Gabriel Pinheiro Modolo

**Affiliations:** 1 Universidade Estadual Paulista – UNESP, São Paulo, SP, Brasil.; 2 Universidade Federal do Triângulo Mineiro – UFTM, Uberaba, MG, Brasil.; 3 Instituto de Diagnóstico Sorocaba, Sorocaba, SP, Brasil.; 4 Academia Brasileira de Neurologia – ABN, Departamento Científico em Reabilitação Neurológica, São Paulo, SP, Brasil.

**Keywords:** neurofibromatosis type 1, fibromuscular dysplasia, stroke, neurofibromatose tipo 1, displasia fibromuscular, acidente vascular encefálico

## Abstract

Neurofibromatosis Type 1 (NF1) is a rare cause of ischemic stroke (IS) in the general population. We report a case of a young patient with NF1 in whom IS was caused by fibromuscular dysplasia. An angiographic study demonstrated occlusion in the right internal carotid artery (ICA), just after its origin, and the left ICA, just before the intracranial portion, and brain magnetic resonance imaging showed the limits of an area of brain infarction in the right frontoparietal region. Despite these concomitant neuroimaging findings, this association is rare, and it is difficult to establish the contribution to the outcome made by each of these diseases, which treatment is the best to implement, or what prognosis is.

## Resumo

A neurofibromatose tipo 1 (NF1) é uma causa rara de acidente vascular cerebral isquêmico (AVCi) na população geral. Neste estudo, relatamos o caso de um paciente jovem com AVCi, com diagnóstico de NF1 associada a displasia fibromuscular. O estudo angiográfico demonstrou oclusão da carótida interna direita, logo após sua origem, e esquerda, antes da porção intracraniana. A ressonância magnética do encéfalo mostrou delimitação de um infarto na região frontoparietal direita. Apesar desses achados concomitantes na neuroimagem, essa associação é rara, sendo difícil de estabelecer a contribuição de cada uma dessas doenças no desfecho, tampouco qual o melhor tratamento a ser implementado e qual o prognóstico.

## INTRODUCTION

Neurofibromatosis Type 1 (NF1) is an autosomal dominant mutation of neurofibromin 1, affecting one in every 2,600-3,000 individuals.^1^ The classical feature of NF1 is development of benign neurofibromas, which are mixed tumors composed of all cell types found in the normal peripheral nerve, and nontumoral manifestations, such as abnormal skin pigmentation (café-au-lait spots), learning disabilities, skeletal abnormalities, and visual anomalies.^2^

NF1 is a rare case of ischemic stroke, since only 6% of patients suffer from cerebral arteriopathies,^2^ while ischemic stokes are more common than hemorrhagic presentations.^3^ Vasculopathy in NF1 remains an under-recognized phenomenon, in particular with regard to its association with increased risk of intracranial aneurysms and moyamoya arteriopathy.^4^,^5^ Acquired degenerative changes and congenital factors have been associated with formation of multiple cerebral aneurysms and could be a risk factor due to vulnerability of the vessel walls.^4^ Intrinsic lesions of the arterial wall are important manifestations of NF-I. More often, the histologic feature is fibromuscular dysplasia with a predominance of intimal thickening.^6^

Given the prevalence of vasculopathy and cerebrovascular anomalies in NF1, an elevated risk of stroke has been highlighted in these patients.^5^ Although in recent years, cerebrovascular disease in NF1 has been better studied and diagnosed, fewer cases have been reported in the last decade. Based on this context, we report a case of a young female patient with stroke, NF1, and no other comorbidities who was also diagnosed with fibromuscular dysplasia (FMD) as an incidental finding during investigation. Our aim is to present this rarely considered link between NF1 and FMD.

## CASE DESCRIPTION

**Figure 1:**
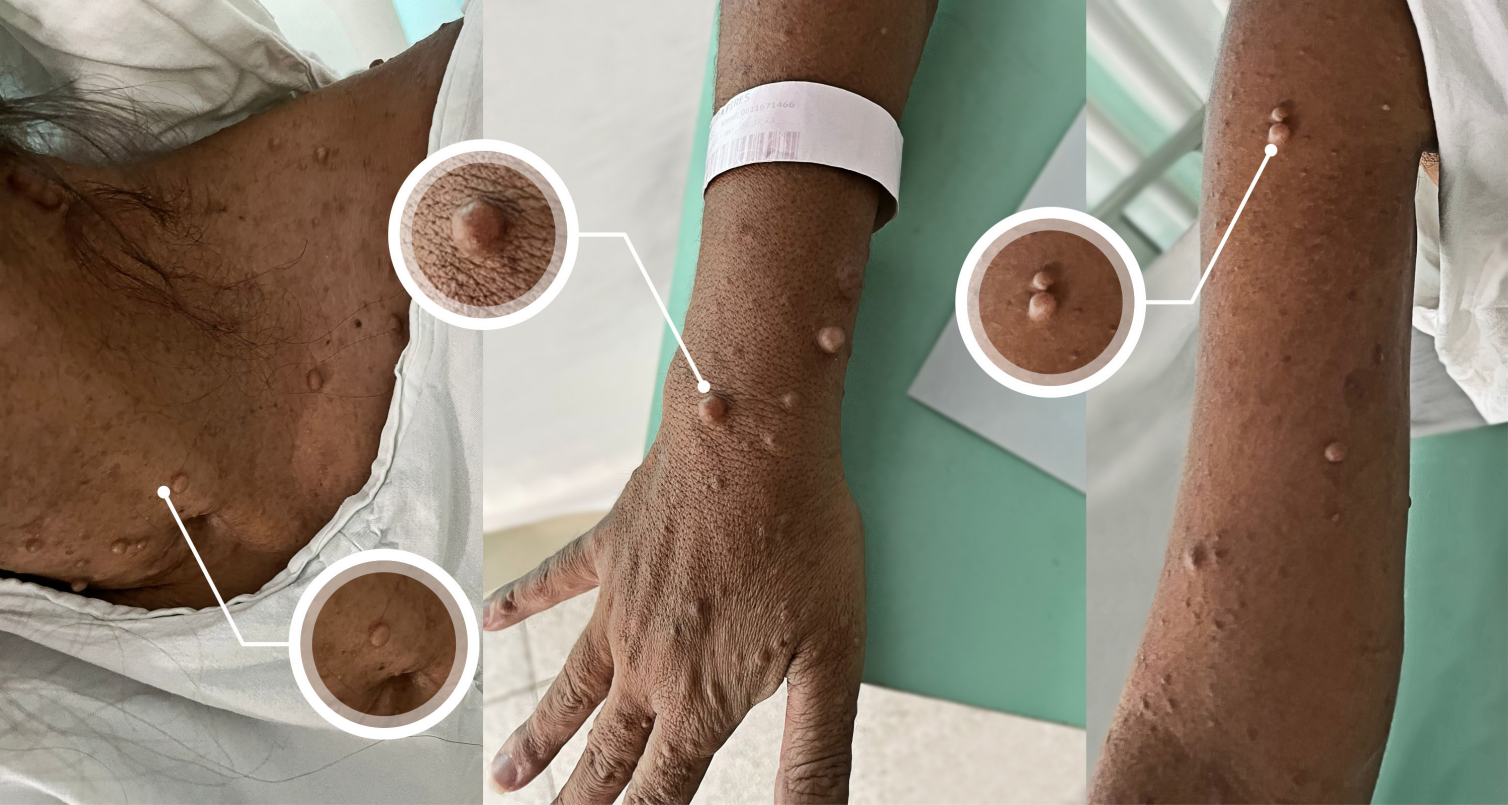
Multiple café-au-lait spots and plexiform neurofibromas in a patient with Neurofibromatosis Type 1.

A 36-year-old woman presented to the emergency room in November 2021 with central left-side facial palsy, hypoesthesia of the left arm and leg, and dysarthria. Cranial computed tomography showed right-side parieto-frontal hypodensity, adjacent to sparce hyperdense lesions, suggesting petechial transformation. The patient was therefore diagnosed with stroke and admitted to the stroke unit. There were no reports of convulsions or signs of infection. The patient had a prior diagnosis of NF1 (multiple café-au-lait spots, plexiform neurofibromas, and freckling in the axilla; Figure 1) and had been prescribed carbamazepine for symptomatic epilepsy after childhood surgery for brain tumors (unknown), without other comorbidities.

**Figure 2:**
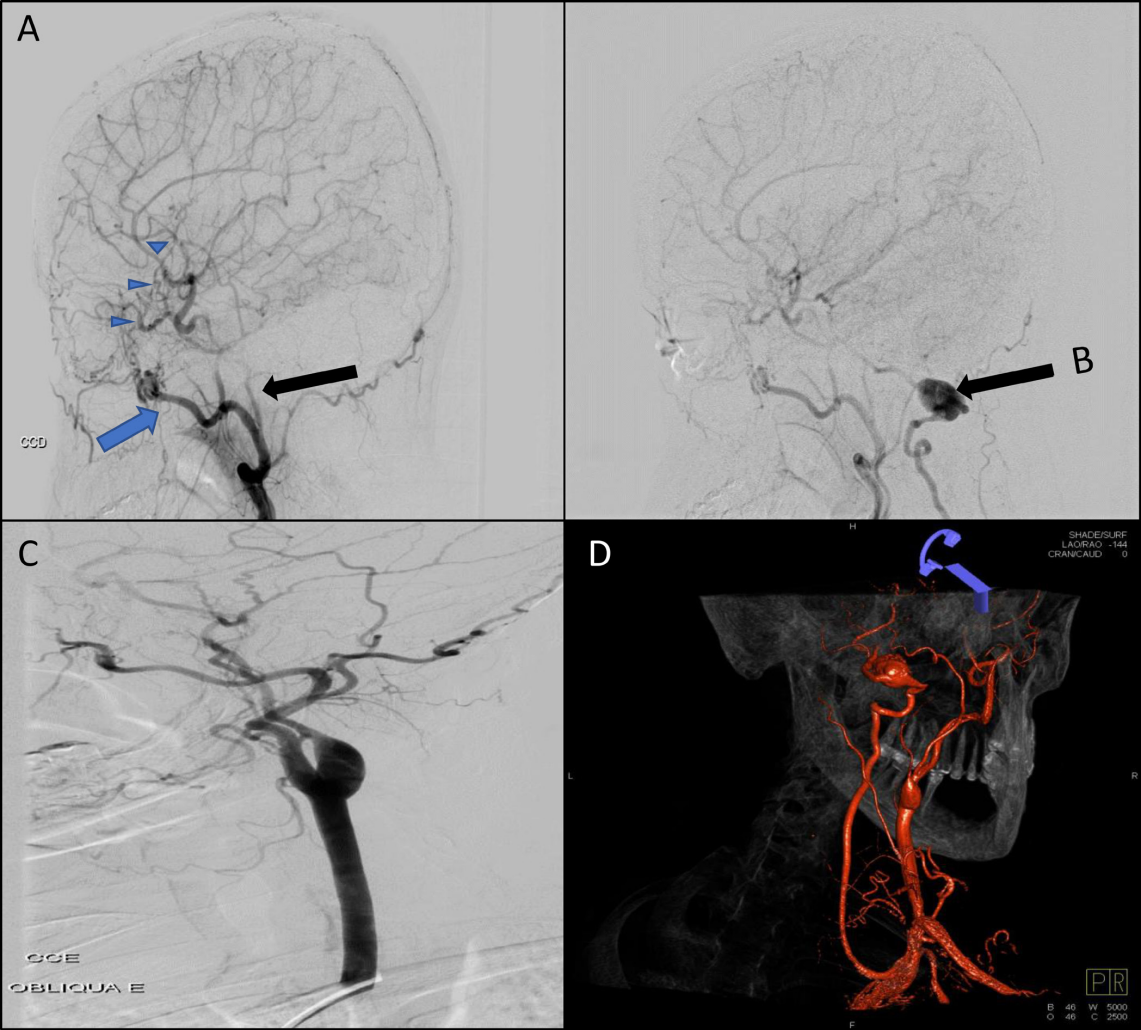
**(a)** Arrowheads: Extra and intracranial collateral circulation; black arrow: severe stenosis of the ICA; blue arrow: maxillary artery; **(b)** vertebral artery pseudoaneurysm; **(c)** fibromuscular dysplasia; **(d)** 3D reconstruction of vertebral artery pseudoaneurysm.

**Figure 3:**
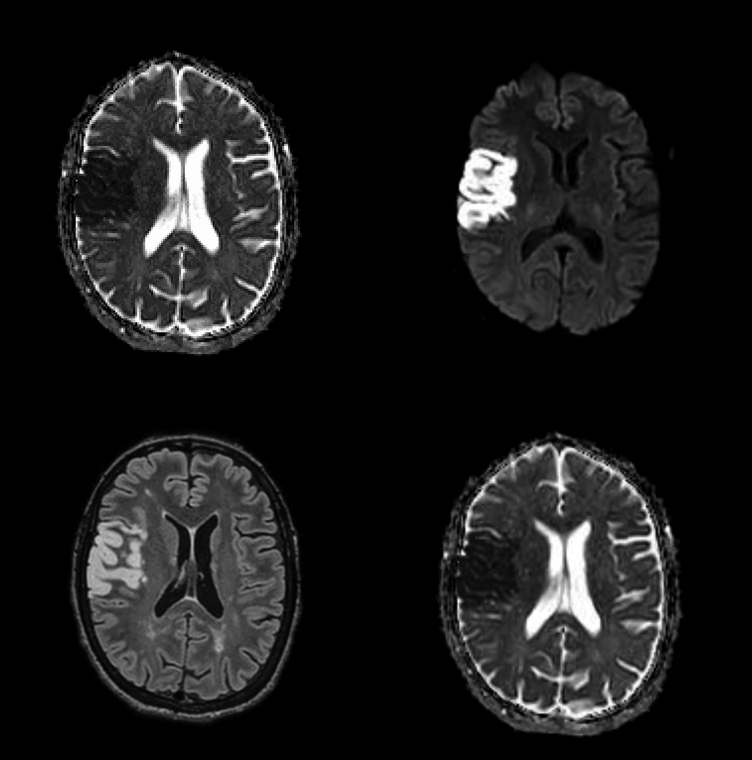
MRI showing a brain infarction in the right frontoparietal region.

The angiographic study demonstrated occlusion in the right internal carotid artery (ICA) soon after its origin, left ICA stenosis after its origin, with occlusion immediately before the intracranial portion. A pseudoaneurysm was also observed in the extracranial portion of the right vertebral artery (Figure 2). Prophylaxis was initiated with 200 mg of acetylsalicylic acid (ASA) and 40 mg of atorvastatin and magnetic resonance imaging (MRI) was ordered. Brain MRI showed the limits of an area of brain infarction in the right frontoparietal region (Figure 3).

The patient underwent arteriography, which showed signs of arterial dissection of the right ICA, with chronic compensation of the left external carotid artery (ECA) and multiple signs of collateral circulation to supply encephalic tissue, probably secondary to previous chronic ICA stenosis, which had since been dissected. The ICA and ECA were stenosed after their origin and in the posterior circulation. There was evidence of a pseudoaneurysm in the extracranial vertebral artery measuring 5 cm wide x 5cm high. She was therefore diagnosed with probable FMD, based on the arteriography findings. Laboratory tests were negative for systemic lupus erythematosus and rheumatoid arthritis (anti-nuclear factor, anti-native DNA, anti-Smith, anti-Ro, anti-La, rheumatoid factor), antiphospholipid syndrome (APS; anticardiolipin antibody lupus anticoagulant, beta-2 microglobulin), and thrombophilia (factor 5 Leiden, antithrombin, prothrombin gene mutation, protein C and S) in 2 different samples taken at a 30-day interval. All imaging and laboratory examinations were performed while the patient was in hospital.

During hospitalization, the patient was stricken with subsegmental pulmonary embolism, with minor clinical repercussions, prompting changes to prophylaxis, from 200 mg ASA to therapeutic anticoagulation with enoxaparin (subcutaneous low-molecular-weight heparin, 60 mg, 12/12h during hospitalization), planning to transition to warfarin on hospital discharge. The patient remained hospitalized for 45 days. At hospital discharge (January, 2022), the patient had central facial palsy and was rated grade 3 modified Rankin scale (mRS). She was followed up for 90 days after hospital discharge (April, 2022) and maintained moderate functional deficit (mRS 3). This study was reviewed and approved by all authors (number: 1.971.819). The patient provided written informed consent to participation in this study.

## DISCUSSION

Hospitalized patients with NF1 have an increased risk of stroke, which affects younger individuals more than the general population.^5^ Clinical manifestations are heterogeneous in younger patients, demonstrating the condition’s potential to provoke changes to cerebral vasculature.^7^,^8^ Barreto-Duarte et al.^9^ have shown that the main site of vascular changes is the ICA and middle and anterior cerebral artery. There are few associations between NF1 and extracranial involvement,^1^ but cranial/intracranial artery involvement is not rare.^10^

The pathogenesis of the vascular lesions in NF1 has not yet been defined. Oderich et al.^11^ demonstrated that the histologic features of younger patients with NF1 were similar to FMD, with predominance of intimal thickening. Vascular lesions in NF1 patients aged ≤ 50 years differed in the predominance of vascular lesions (aortic, renal, mesenteric, and carotid-vertebral stenosis or aneurysms). Moreover, the histological findings in these younger patients showed FMD, in striking contrast to the degenerative atherosclerotic changes seen in older patients.

The prognosis for NF1-related stroke is favorable in young populations with adequate rehabilitation;^3^ however, children usually have worse outcomes.^9^ The rate of recurrence of such strokes is still unknown. FMD is a rare systemic vascular disease; commonly affecting the renal and carotid arteries. However, intracranial progression occurs in approximately 8% of cases.^5^ This can be asymptomatic or may cause arterial dissections or intracranial aneurysms. Diagnosis is based on consistent findings from diagnostic imaging, since histopathology is no longer part of the diagnosis.^12^

The association between FMD and NF1 is a very rare cause of stroke. In this case, we saw a patient with NF1 who appears to have had carotid FMD without renal artery involvement. Even if it is clear that NF1 is associated with intrinsic vascular lesions, the angiographic findings in this case were consistent with the hypothesis of FMD in the context of NF1. Despite the lack of clinical criteria, differential diagnosis to rule out APS is still important in this case. However, the latest consensus recommends a minimum of a 12-week interval between laboratory tests to confirm APS.^13^ Therefore, technically, APS cannot be ruled out and the patient is being followed up at an outpatient dermatology clinic for further tests.
